# Comparison of erector spinae plane block and serratus anterior plane block for postoperative analgesia in uniportal thoracoscopic lobectomy: a randomized controlled trial

**DOI:** 10.1186/s12871-023-02353-0

**Published:** 2023-12-01

**Authors:** Wei Wu, Huan Xu, Xue Chen, Wenxin He, Hong Shi

**Affiliations:** 1 Department of Anesthesiology, Shanghai Pulmonary Hospital, School of Medicine, Tongji University, Shanghai, 200433 China; 2 Department of Thoracic Surgery, Shanghai Pulmonary Hospital, School of Medicine, Tongji University, Shanghai, 200433 China

**Keywords:** Erector spinae plane block, Serratus anterior plane block, Postoperative pain, Uniportal thoracoscopic surgery

## Abstract

**Background:**

Postoperative pain remains a significant concern following uniportal thoracoscopic surgery. The analgesic efficacy of erector spinae plane block (ESPB) and serratus anterior plane block (SAPB) in terms of postoperative opioid consumption in uniportal thoracoscopic surgery still needs further studies.

**Methods:**

A randomized controlled trial was conducted, enrolling 150 patients who underwent uniportal thoracoscopic lobectomy. The patients were randomly allocated to three groups in a 1:1:1 ratio: the ESPB group (administered 20 ml of 0.5% ropivacaine), the SAPB group (administered 20 ml of 0.5% ropivacaine), and the standard care (control) group. The primary endpoint was the consumption of sufentanil during the first 24 h following surgery. Secondary endpoints assessed the area under the curve (AUC) of pain numerical rating scale (NRS) scores, occurrence of moderate to severe pain, time to initial sufentanil request, and postoperative adverse events.

**Results:**

No significant difference was observed in the consumption of sufentanil during the first 24 h following surgery between the ESPB and SAPB groups (adjusted difference, 1.53 [95% CI, -5.15 to 2.08]). However, in comparison to the control group, both intervention groups demonstrated a significant decrease in sufentanil consumption, with adjusted differences of -9.97 [95% CI, -13.10 to -6.84] for the ESPB group and -12.55 [95% CI, -15.63 to -9.47] for the SAPB group. There were no significant differences in AUC of NRS scores during rest and movement between the ESPB and SAPB groups, with adjusted differences of -7.10 [95% CI, 1.33 to -15.55] for the rest condition and 5.61 [95% CI, -13.23 to 2.01] for the movement condition. At 6 h postoperatively, there were fewer patients with moderate to severe pain in the ESPB group compared with those in the SAPB group (adjusted difference, -1.37% [95% CI, -2.29% to -0.45%]. The time to first sufentanil request significantly differed among the three groups (ESPB vs Control *P* < 0.01, SAPB vs Control *P* < 0.01, ESPB vs SAPB *P* = 0.015).

**Conclusions:**

In patients undergoing uniportal thoracoscopic lobectomy, although the differences between the two groups are not statistically significant, both the ESPB and SAPB demonstrate effective reduction in postoperative opioid consumption and the need for rescue analgesics compared to the control group. Moreover, the ESPB group experienced a significantly lower incidence of moderate to severe pain at 6 h postoperatively compared to the SAPB group.

**Trial registration:**

The study was registered in the Chinese Clinical Trial Registry (registration No: ChiCTR1900021695, Date of registration: March 5th, 2019).

**Supplementary Information:**

The online version contains supplementary material available at 10.1186/s12871-023-02353-0.

## Background

The advancement of surgical expertise and ongoing developments in surgical instrumentation have led to the increasing popularity of video-assisted thoracoscopic surgery (VATS) in the management of lung cancer [1, 2]. Uniportal VATS, a novel minimally invasive surgical technique utilizing a single small incision for chest cavity surgery, has emerged as a promising alternative [3]. Compared to the traditional multiport VATS approach, uniportal VATS offers several advantages such as decreased postoperative pain, shorter hospitalization and enhanced cosmetic outcomes [4–7].

However, it's essential to recognize that postoperative pain management remains a critical concern [8, 9]. Inadequate pain control can have a detrimental impact on recovery quality, potentially increasing the risk of postoperative pulmonary complications and thromboembolic events, leading to prolonged hospital stay [10, 11]. Therefore, optimizing acute postoperative analgesia for patients undergoing uniportal VATS is of paramount importance.

Currently, various regional blockade techniques are available for thoracic surgery, including thoracic paravertebral block, serratus anterior plane blocks (SAPB), and erector spinae plane blocks (ESPB) [12]. Among these, SAPB and ESPB have gained popularity for VATS due to their technical simplicity and safety profile [13]. However, they differ in terms of their extent of blockade. ESPB affects both the dorsal and ventral rami of the thoracic spinal nerves, leading to some degree of sympathetic blockade, while SAPB specifically targets the lateral cutaneous branches of the thoracic intercostal nerves, providing sensory blockade that covers the T2-T9 dermatomes [14–16].

Despite their widespread use, the analgesic efficacy of ESPB and SAPB in VATS surgery remains a topic of debate, as multiple RCTs and meta-analyses have reported conflicting results [13, 17–24]. Moreover, uniportal VATS does not involve rib spreading and results in significantly less trauma compared to multiport VATS and open surgery [25, 26]. Currently, there is a lack of research directly comparing the effectiveness of ESPB and SAPB for pain relief after uniportal VATS. Hence, our study aims to evaluate and compare the analgesic efficacy of ESPB, SAPB, and blank control in patients undergoing uniportal VATS through a randomized controlled trial. Our hypothesis suggests that both ESPB and SAPB offer superior pain relief for patients undergoing uniportal VATS compared to the control group, with similar analgesic efficacy between the ESPB and SAPB groups.

## Methods

### Trial design

This prospective, randomized, 3-arm, observer-blinded, controlled trial was conducted to compare the efficacy of analgesia between ESPB and SAPB in patients undergoing uniportal VATS. The study was approved by the Ethics Committee of Shanghai Pulmonary Hospital (reference number K19-006), and registered at the Chinese Clinical Trial Registry on March 5th, 2019 (reference number ChiCTR1900021695). Written informed consent was obtained from all eligible patients prior to enrollment, upon their arrival in the operating room for the scheduled surgery.

### Eligible patients

The target population consisted of patients aged 18 to 79, scheduled for elective uniportal VATS lobectomy, with an American Society of Anesthesiologists (ASA) physical status of I or II and normal lung function (FEV1/FVC ≥ 70%). Exclusion criteria included infection at the puncture site, body mass index (BMI) > 40, severe heart disease or renal insufficiency, thoracic deformity, allergy to local anesthetics, the presence of a neuromotor disorder or mental illness, and patients who did not accept to participate.

### Randomization and masking

Participants were randomly assigned in a 1:1:1 ratio to the ESPB group, SAPB group, or control group utilizing block randomization with block sizes of 6. Random numbers generated by Stata 15.0 (StataCorp LLC, 4905 Lakeway Drive) were used for allocation. Sealed, opaque, sequentially numbered envelopes were used to ensure allocation concealment. While it was not feasible to mask the anesthesiologist performing the ultrasound-guided regional nerve blocks and participants themselves, efforts were made to minimize potential bias in postoperative care by not informing other care providers involved in the study about the allocated intervention. Outcome assessors and data analysts remained masked to treatment allocations.

### Study intervention

Upon entering the anesthetic induction room, patients underwent regular monitoring including electrocardiogram, heart rate, blood oxygen saturation and non-invasive blood pressure. The nerve blocks were administered approximately 30 mins before the induction of general anesthesia. Patients receiving ESPB and SAPB were not routinely sedated. Skilled consultant anesthesiologists, experienced in ESPB and SAPB, performed or supervised the procedures. Regardless of the specific block performed, each patient received 20 ml of 0.5% ropivacaine. The blocks were performed using a 20-gauge echogenic needle (001156–72, PAJUNK, USA) and an ultrasound machine equipped with a linear ultrasound transducer (L38Xi, Sonosite,USA).

#### ESPB procedure

For participants assigned to the ESPB group, the unilateral block was performed in the prone position following the technique described by Chin K et al. [27]. Prior to the procedure, the skin was prepared using 10% povidone iodine. The block was carried out at the T5 level of the spine. Once the correct transverse process (TP) was identified, a 20-gauge needle was inserted using an in-plane, cranial-to-caudad approach, placing the tip within the fascial plane on the deep aspect of the erector spinae muscle. The accuracy of needle tip placement was confirmed by administering 0.5–1 ml of local anesthetic. Subsequently, 20 ml of 0.5% ropivacaine was administered.

#### SAPB procedure

For participants assigned to the SAPB group, the unilateral block was performed in the lateral decubitus position, following the technique described by Blanco et al. [16]. After preparing the skin, a high-frequency transducer was positioned along the participant's midaxillary line in the transverse plane at the level of the fifth rib. The needle was inserted in-plane, targeting the plane deep to the serratus anterior muscle. Continuous ultrasound guidance was used to ensure precise needle placement. Subsequently, a 20 ml bolus of 0.5% ropivacaine was injected, with careful aspiration to confirm the absence of air or blood prior to administration.

#### Control group procedure

Participants assigned to the control group did not undergo any placebo or sham procedures.

After the nerve block was completed, patients were transferred to the operating theater. General anesthesia was induced with propofol (1.5–2 mg/kg), sufentanil (0.3–0.4 mcg/kg), midazolam (0.02 mg/kg), and cisatracurium (0.3 mg/kg). Following intubation with a double-lumen endotracheal tube (DLT), anesthesia was maintained with propofol and remifentanil to achieve a spectral entropy value between 40 and 60. We selected the appropriate left or right-sided DLT based on the surgical side and confirmed the placement of the DLT using bronchoscopy. A one-lung protective ventilation strategy was implemented, characterized by tidal volumes of 6 mL/kg or lower based on predicted body weight, and a positive end-expiratory pressure of 5–10 cmH_2_O. Following extubation in the operating room, participants were subsequently transferred to the Post-Anesthesia Care Unit (PACU). Intravenous dexamethasone (5 mg) and tropisetron (5 mg) were administered prior to the induction of general anesthesia as prophylaxis against postoperative nausea and vomiting (PONV).

#### Surgical procedure

During the procedure, a 3.5 cm incision was made in the anterior axillary line at the 4th intercostal space for upper lobe resection and at the 5th intercostal space for middle and inferior lobe resection. A thoracoscope was used along with specific surgical instruments and either harmonic shear or hook electro-cautery. The bronchus, vein, and artery were dissected separately using an endolinear stapler or ligated with hem-o-loks. The specimen was then placed in a specimen bag. Subsequently, the surgeons sutured the incision after sufficient hemostasis.

Postoperative pain management followed a standardized protocol for all patients. This included a combination of 5 mcg sufentanil and 50 mg flurbiprofen axetil administered 30 mins before the surgery ended, as well as a daily intravenous infusion of 50 mg flurbiprofen axetil for postoperative analgesia. Patient-controlled analgesia (PCA) was implemented using a 24-h infusion of sufentanil 1 mcg/ml solution. The PCA protocol included an infusion rate of 2 ml/h, a 2 ml bolus dose, a lockout time of 15 mins, and a maximum limit of 10 ml/h. The criterion for initiating PCA treatment was a numerical rating scale (NRS) score > 2. Oxycodone 5 mg/acetaminophen 325 mg was available as a rescue analgesic agent. Intravenous tropisetron at a dose of 5 mg was administered in the hospital ward for the management of PONV.

### Study endpoints

The primary endpoint was the total consumption of sufentanil during the initial 24 h postoperatively, recorded from the PCA device. Secondary endpoints included: (1) Area under the curve (AUC) of NRS scores for pain at rest and on movement over a 24-h period. (2) Time to the first administration of sufentanil analgesia. (3) Incidence of postoperative opioid side effects (nausea, vomiting, and dizziness) and complications. (4) Participant satisfaction with the effectiveness of analgesia during the initial 24 h postoperatively was rated on a 5-point Likert scale, ranging from 'highly unsatisfactory' to 'highly satisfactory' [28].

### Statistical analysis

The sample size was calculated using PASS Software version 15 for the analysis of variance (ANOVA) test to detect differences in sufentanil consumption during the first 24 h postoperatively among the three groups. Based on an unpublished preliminary study conducted at the institution, the mean (standard deviation) sufentanil consumption in the ESPB group, SAPB group, and control group was estimated to be 41 (12), 44 (16), and 55 (22), respectively. With a statistical power of 80% and a significance level (α) of 0.05, a minimum of 44 patients per group was calculated. Accounting for a 10% dropout rate, the planned total sample size was 150 patients, with 50 subjects allocated to each group.

Intention-to-treat (ITT) analyses were performed to assess the effect of the intervention on all participants. Statistical analysis was conducted using Stata 15.0 software. Linear regression with robust standard errors adjusted for surgical doctors was used to analyze the primary endpoint. For the analysis of secondary endpoints related to pain, linear regression and logistic regression models were employed, adjusting for preoperative pain, age, BMI, and history of smoking [29]. The log-rank test was used to compare the time to the first administration of sufentanil analgesia. To account for multiple comparisons, the Bonferroni correction was applied, resulting in a significance threshold of *p* = 0.05/3 = 0.0167.

## Results

### Study participants and enrollment

**Fig. 1:**
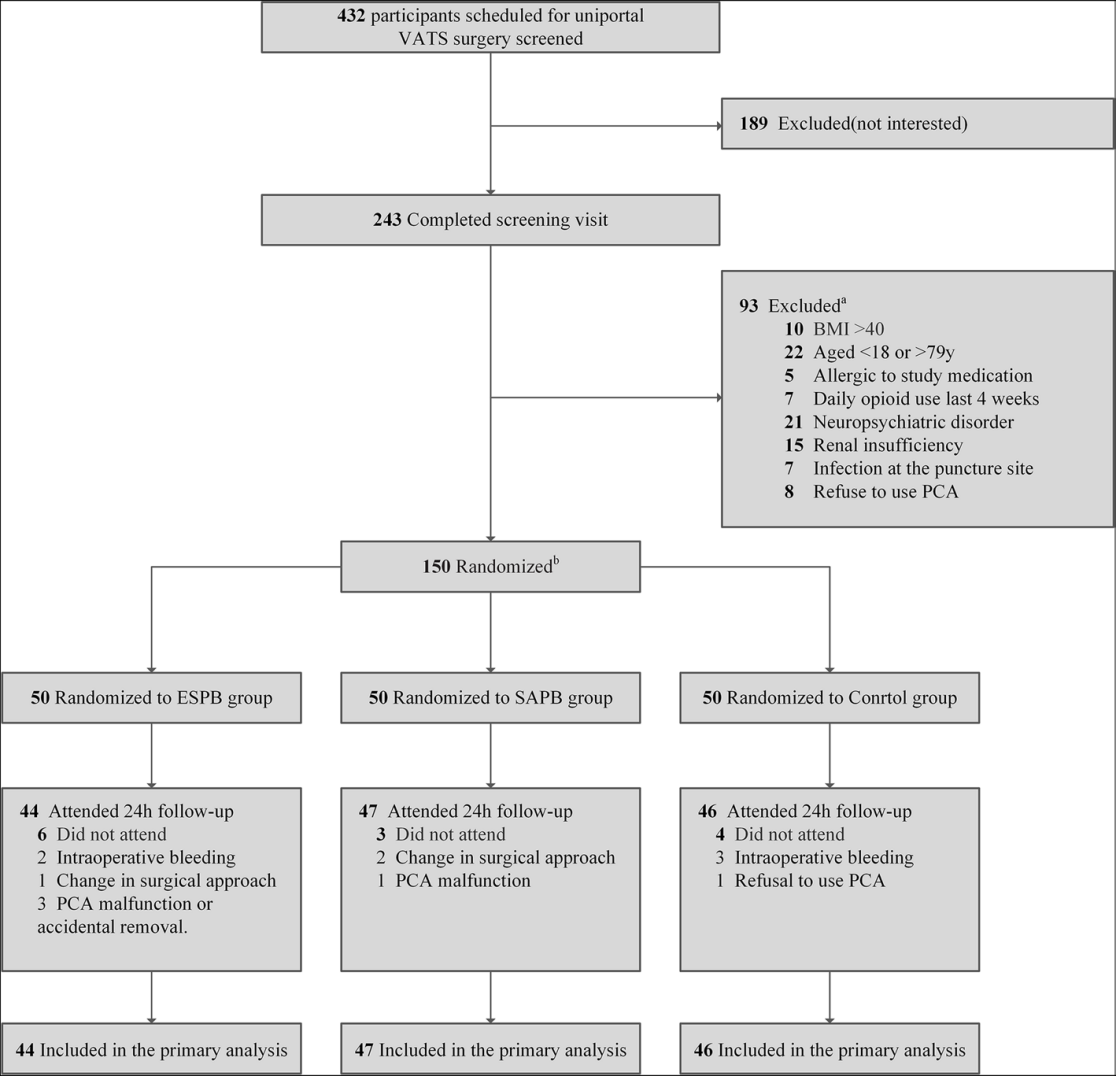
Participant flow through the study. Abbreviations: ESPB, erector spinae plane blocks; SAPB, serratus anterior plane blocks. **a** Participant may have been ineligible for more than 1 reason. **b** Block randomization method was used to assign all eligible persons to 1 of 3 intervention groups

From January 2019 to January 2022, a total of 150 patients went randomization. After excluding 13 patients, the final analysis included 137 patients (Fig. 1), The mean (SD) age of the participants was 57(12) years, and there were 57 male patients. The number of patients who withdrew from the trial was comparable across the three groups, with 6 patients in the ESPB group, 3 patients in the SAPB group, and 4 patients in the control group. The study groups were also well matched in terms of demographic and baseline characteristics, except for the ESPB group, which had longer durations of surgery and anesthesia (Table 1).

**Table 1:** Demographic and clinical characteristics of participants at baseline

**Baseline characteristics**	**Treatment group**, **No. (%)**		**Control**
	**ESPB**	**SAPB**	
No	44	47	46
Age, mean (SD), y	58(10)	55(13)	56(11)
Height, mean (SD), cm	163(8)	164(8)	162(8)
Weight, mean (SD), kg	63(8)	62(9)	64(10)
BMI, mean (SD)	23.8(2.6)	23.0(3.4)	24.3(3.2)
Sex			
Women	26(60)	26(55)	28(61)
Men	18(40)	21(45)	18(39)
ASA			
I	21(48)	22(47)	22(48)
II	23(52)	25(53)	24(52)
Smoking status			
Non-smoker	32(73)	31(66)	33(72)
Smoker	12(27)	16(34)	13(28)
Hypertension	17(38)	14(29)	13(28)
Cardiovascular disease	5(11)	7(15)	5(11)
Diabetes	6(14)	5(11)	10(22)
Preoperative pain ^a^	6(14)	4(9)	3(7)
Duration of surgery, mean (SD), min	106.7(63.0)	82.1(41.0)	98.9(50.5)
Duration of anaesthesia, mean (SD), min	123.7(65.0)	102.5(39.9)	113.5(48.7)
Location of port (intercostal space)			
4	21(48)	19(40)	24(52)
5	23(52)	28(60)	22(48)
Procedure			
LU	17(39)	11(23)	8(17)
LL	3(7)	7(15)	4(9)
RU	17(39)	19(40)	19(41)
RM	1(2)	4(9)	5(11)
RL	6(13)	6(113)	10(22)
Number of chest tubes			
1	20(45)	30(64)	31(67)
2	24(55)	17(36)	15(33)

*Abbreviations*: *BMI* body mass index, calculated as weight in kilograms divided by height in meters squared, *ASA* American Society of Anesthesiologists physical status, *LU* Left upper lobectomy, *LL* Left lower lobectomy, *RU* Right upper lobectomy, *RM* Right Middle lobectomy, *RL* Right lower lobectomy

^a^ Preoperative pain status is defined as the existence of pain before surgery, including pain induced by lung diseases and comorbidities, with a NRS (Numeric Rating Scale) score of more than 3

### Primary endpoint

**Fig. 2:**
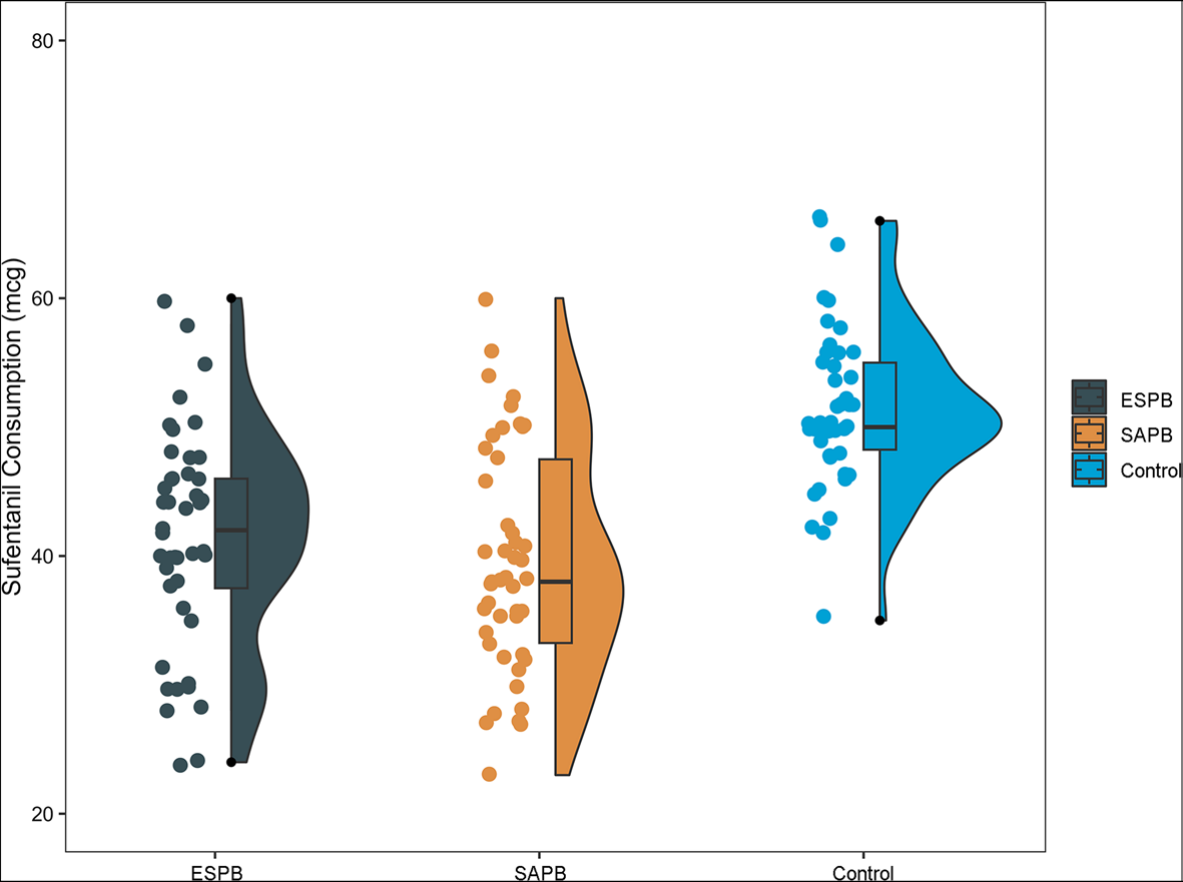
Sufentanil consumption at 24 h postoperatively among the three groups. Abbreviations: ESPB, erector spinae plane blocks; SAPB, serratus anterior plane blocks. The middle line in the plot boxes represents the median values, while the boxes indicate the interquartile range. The whiskers extend to the most extreme observed values within 1.5 times the interquartile range of the nearer quartile

There was no significant difference in postoperative 24-h sufentanil consumption between the ESPB and SAPB groups (adjusted difference, 1.53 [95% CI, -5.15 to 2.08]). However, both the ESPB and SAPB groups showed a significant decrease in postoperative 24-h sufentanil consumption compared to the control group. The adjusted differences were -9.97 [95% CI, -13.10 to -6.84] for the ESPB group and -12.55 [95% CI, -15.63 to -9.47] for the SAPB group (Fig. 2 and Supplementary Material 1).

### Secondary endpoint

**Fig. 3:**
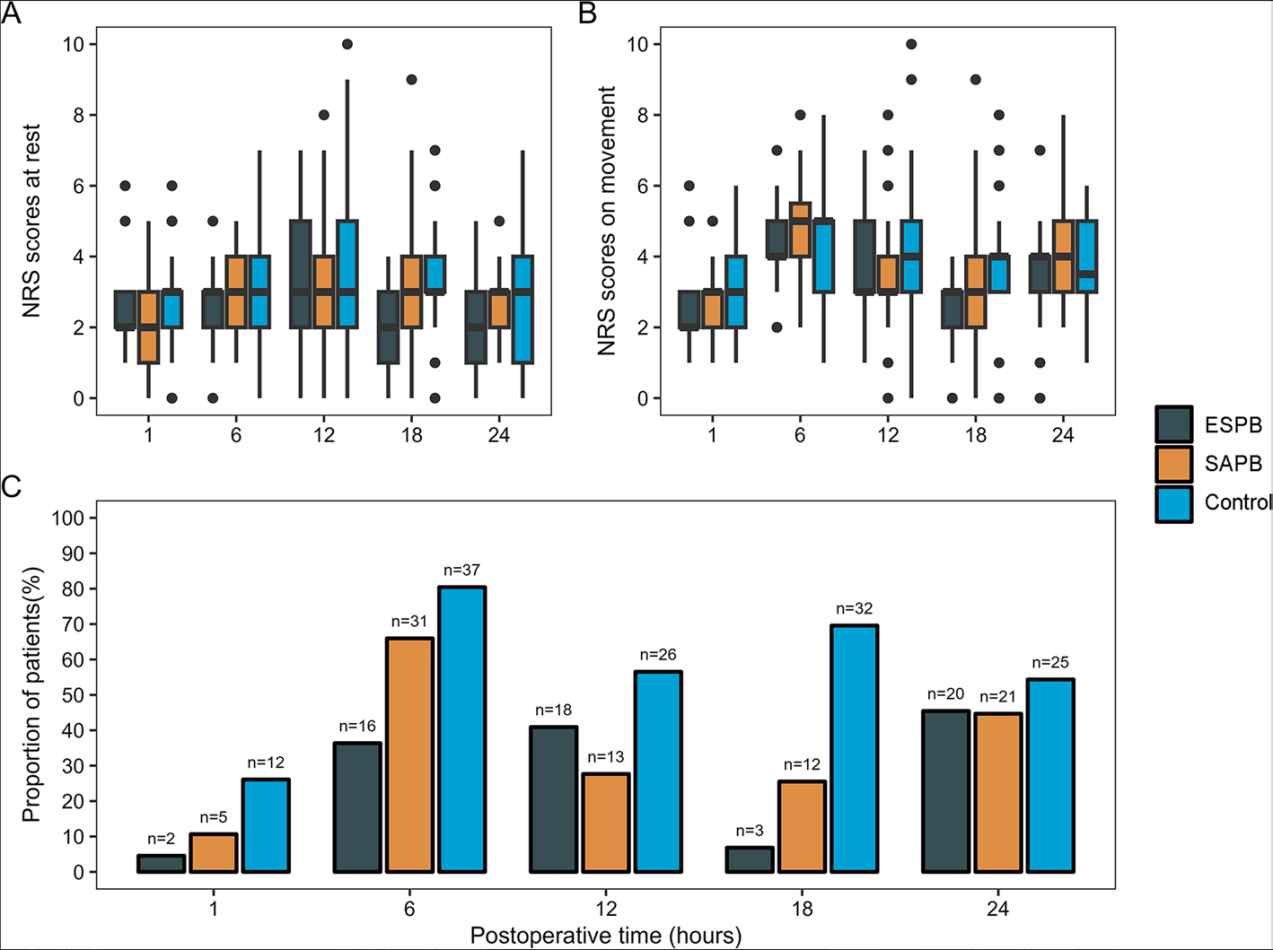
The pain scores and the proportion of patients experienced moderate-to-severe pain during follow-up. Abbreviations: ESPB, erector spinae plane blocks; SAPB, serratus anterior plane blocks. The middle line in the plot boxes represents the median values, while the boxes indicate the interquartile range. The whiskers extend to the most extreme observed values within 1.5 times the interquartile range of the nearer quartile and the dots represent observed values outside the range. Moderate-to-severe pain was defined as a numeric rating scale score of 3 or higher. **A** Numeric Rating Scale (NRS) scores at rest during follow-up. **B** Numeric Rating Scale (NRS) scores on movement during follow-up. **C** Proportion of patients with moderate-to-severe postoperative pain during follow-up

No significant differences in AUC of NRS scores for pain vs time were observed between the ESPB and SAPB groups, both during rest and movement. The adjusted differences were -7.10 [95% CI, 1.33 to -15.55] for the rest condition and 5.61 [95% CI, -13.23 to 2.01] for the movement condition (Fig. 3A, B, Supplementary Material 1 and additional file 1).

Both the ESPB and SAPB groups had a lower number of patients requiring rescue analgesia compared to the control group, but no significant difference was found between the ESPB and SAPB groups. In the three groups, there were variations in the number of patients experiencing moderate-to-severe pain at different time intervals, except at 24 h postoperatively. At 6 h postoperatively, there were fewer patients with moderate to severe pain in the ESPB group compared with those in SAPB group (adjusted difference, -1.37% [95% CI, -2.29% to -0.45%] (Fig. 3C and Supplementary Material 1).

**Fig. 4:**
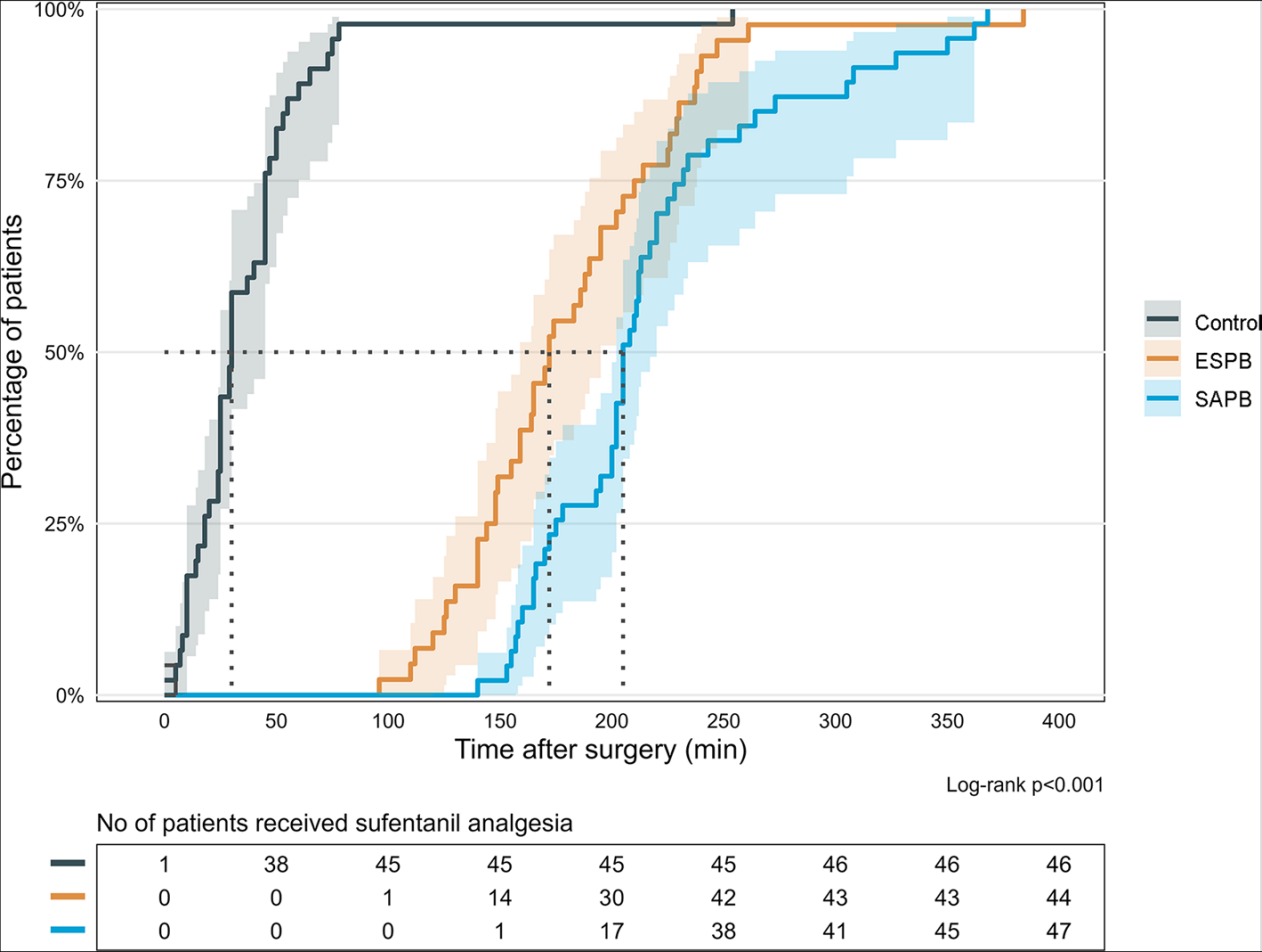
The cumulative probability of the initial need for sufentanil analgesia during follow-up. Abbreviations: ESPB, erector spinae plane blocks; SAPB, serratus anterior plane blocks. The shaded areas represent pointwise 95% CIs for each treatment group. The pairwise comparisons among the three groups were conducted using the log-rank analysis

The time to initial administration of sufentanil analgesia showed significant differences among the three groups, as indicated by log-rank analysis for comparison (ESPB vs Control *P* < 0.01, SAPB vs Control *P* < 0.01, ESPB vs SAPB *P* = 0.015) (Fig. 4). The SAPB group had the longest median (IQR) time to first sufentanil analgesia, with a duration of 205 (202–220) minutes, followed by the ESPB group with 172 (159–195) minutes, and the control group with 30 (25–45) minutes.

The adverse events observed during the study are summarized in Table 2. Among the ESPB, SAPB, and control groups, 9, 12, and 7 cases of postoperative complications were reported, respectively. Additional, neither the ESPB group nor the SAPB group experienced any complications related to nerve blockade.

**Table 2:** Adverse Events in the safety population in the study^a^

Characteristics	**Treatment group**, **No. (%)**			
	**ESPB**	**SAPB**	**Control**	***P* value**^**b**^
No	50	50	50	
Hypotension in PACU^c^	2(4)	2(4)	1(2)	0.81
Reported block complications^d^	0	0		0.99
PONV^e^	5(10)	4(8)	6(12)	0.80
Dizziness	1(2)	2(4)	3(6)	0.59
No. of patients with ≥ 1 postoperative complications	9(18)	12(24)	7(15)	0.43
Pneumonia	3(6)	7(14)	4(5)	0.36
Recurrent pneumothorax/Air leak requiring additional intervention	5(10)	4(8)	3(6)	0.76
Arrhythmia	1(2)	0(0)	0(0)	0.36
Bleeding necessitating blood transfusion	0(0)	1(2)	1(2)	0.60

*Abbreviations*: *PACU* Post-Anesthesia Care Unit, *PONV* postoperative nausea and vomiting

^a^ Safety population comprised patients who were randomized and received at least one treatment from the assigned group

^b^ Chi-square test was employed in analysis

^c^ Hypotension that necessitates pharmacological intervention is typically defined as follows: a systolic blood pressure below 90 mmHg or a decrease of more than 20% from the patient's baseline blood pressure

^d^ Complications associated with nerve blocks can include nerve injury, vascular injury, local anesthetic allergy or hypersensitivity reaction, blockade failure or incomplete blockade, local muscle pain, excessive bleeding, hematoma formation, or local infection

^e^ Postoperative nausea and vomiting is defined as the experience of nausea, vomiting, or both during the first 24 h following surgery

## Discussion

Our study represents the first randomized controlled trial comparing the ESPB and SAPB block in uniportal VATS. To enhance the sensitivity of treatment outcome evaluation, we included a blank control group [30]. Our results demonstrated comparable postoperative sufentanil consumption between the ESPB and SAPB groups. Additionally, there was no significant difference in the AUC for pain scores during rest and activity between the two groups. However, the ESPB group experienced a significantly lower incidence of moderate to severe pain at 6 h postoperatively compared to the SAPB group.

The primary endpoint measure for our study was the postoperative consumption of opioid analgesics. Intravenous or oral opioids are currently the most commonly used methods for managing moderate to severe pain. However, it is important to acknowledge the diverse adverse effects associated with opioid analgesics. Acute side effects, including nausea, itching, respiratory depression, and constipation, are frequently observed with opioid use. These adverse effects not only result in prolonged hospital stays but also have unforeseen consequences such as hospital readmissions, dependency, addiction, the development of opioid-induced hyperalgesia, and the onset of chronic pain [31]. Furthermore, several studies have reported an association between opioid use and tumor recurrence [32, 33]. Additionally, the excessive and inappropriate use of opioids following surgery can contribute to the opioid crisis, encompassing issues such as diversion, misuse, and addiction to opioid analgesics.

In our study, we implemented Patient-controlled Analgesia (PCA) for postoperative pain management, which is considered more effective than standard intravenous opioid painkillers in major surgeries [34]. By allowing patients to self-regulate their pain medication, PCA ensures effective pain relief with minimal side effects [34, 35]. We employed an intermittent, fixed demand dosing (self-administering) with continuous background infusion in the intravenous PCA model. Acknowledging the prevalence of intense post-thoracoscopic pain, we suggested that continuous background infusion doses could better alleviate patient discomfort [34]. Furthermore, recent research has introduced a new oral opioid pain relief method in conjunction with intravenous PCA [36]. This approach is known for its non-invasive nature and low risk of adverse reactions, potentially establishing it as a primary option for pain relief in future thoracic surgeries [36].

In contrast to previous studies focusing on multi-port thoracoscopic and robotic thoracoscopic surgeries [13, 22, 37, 38], our study in uniportal thoracoscopic surgery revealed comparable levels of opioid consumption between ESPB and SAPB blocks. This finding can be primarily attributed to the differences in port location selected for the surgery. In uniportal thoracoscopic surgery, the port is typically positioned at the 4th and 5th ribs, whereas other minimal thoracoscopic surgeries may place the camera port at the 7th-8th intercostal space [39]. Moreover, ESPB blocks exert a sympathetic blockade effect, while SAPB blocks selectively target the intercostal nerve branches. The extent of all interfascial blocks, including SAPB block, is closely associated with the spread of local anesthetics within the tissue plane, which can vary significantly among patients. Consequently, there may be occasions where the range of SAPB block falls short of covering the incision area required for minimal thoracoscopic surgery [40], although this occurrence is significantly reduced in uniportal thoracoscopic surgery. Another contributing factor is the remarkable reduction in postoperative opioid consumption reported in previous studies involving uniportal thoracoscopic surgery when compared to traditional VATS [41], thereby accounting for the narrower differences in opioid consumption between the two groups. Finally, it is important to acknowledge that some studies were not adequately powered to compare opioid consumption [13, 22, 38].

In our study, we evaluated pain scores and the incidence of moderate to severe pain to increase the sensitivity of the results. The higher usage of opioid analgesics in the control group and the background dose of PCA in all three groups may explain the similar area under the curve (AUC) for pain scores during rest among the three groups. However, consistent with multiple previous studies [42, 43], the control group exhibited a significantly higher AUC for pain scores during activity and an increased incidence of moderate to severe pain during follow-up.

Our study revealed a longer time to the initial administration of sufentanil analgesia in the SAPB group compared to the ESPB group. However, it is important to note that the duration of analgesia can vary significantly between SAPB and ESPB in different studies [21, 22, 38], possibly due to variations in the choice of local anesthetic agents, concentrations and volumes used. Additionally, there is currently no consensus on the recommended doses and volumes for ESPB and SAPB. The question of whether the "deep," "superficial," or "combined" SAPB provides superior analgesia remains a topic of ongoing debate [24]. In our center, we opted for the deep block as we considered it to be more easily performed. While intercostal nerve block (ICNB) by the operating surgeon under thoracoscopic guidance is a commonly used postoperative analgesic technique [44], we chose not to use ICNB in the control group to create a distinct comparison between the interventions. Furthermore, earlier studies have indicated that patients receiving continuous ESPB required fewer opioids and reported less pain than those receiving ICNB within the initial 48 h after surgery [45]. However, literature findings concerning SAPB and ICNB demonstrate conflicting results in VATS [17, 18]. Future research might be necessary to investigate the analgesic efficacy of ESPB combined with ICNB compared to SAPB combined with ICNB in uniportal thoracoscopy.

Consistent with prior researches [18, 22], our study demonstrated the absence of any nerve block-related complications in both the ESPB and SAPB groups, indicating the relative safety of these techniques. Our study reported a lower incidence of postoperative complications compared to the results obtained from the European Society of Thoracic Surgeons database [46]. This favorable outcome can be partly attributed to the inclusion of patients with ASA scores of I-II in our study. In addition, it is also worth noting the comprehensive definition of postoperative complications, which encompasses a range of diverse outcomes.

Our study has several limitations. One limitation of our study is that we only evaluated the consumption of sufentanil over a 24-h period. This limited timeframe may not provide a comprehensive understanding of the complete analgesic effect postoperatively. Another limitation is the absence of sensory testing following the nerve block in our study. Furthermore, the use of 20 ml of 0.5% ropivacaine as the local anesthetic might require additional research to determine its optimal use. Additionally, our study was conducted at a single center, which poses limitations on the generalizability of the findings. Consequently, the generalizability and applicability of the study results may be limited and further validation through multicenter studies is warranted.

## Conclusions

In conclusion, our study demonstrated comparable 24-h postoperative sufentanil consumption between the ESPB and SAPB groups in patients undergoing uniportal thoracoscopic lobectomy, with both intervention groups showing significantly lower consumption compared to the control group. Moreover, the ESPB group notably experienced a significantly lower incidence of moderate to severe pain at 6 h postoperatively compared to the SAPB group.

### Supplementary Information

**Additional file 1.**

**Additional file 2. **

## Data Availability

The datasets generated during and/or analyzed during the current study are available from the corresponding author on reasonable request.

## Abbreviations

ESPB: Erector Spinae Plane Block
SAPB: Serratus Anterior Plane Block
VATS: Video-Assisted Thoracoscopic Surgery
RCT: Randomized Controlled Trial
ASA: American Society of Anesthesiologists
PACU: Post-Anesthesia Care Unit
TP: Transverse Process
AUC: Area Under the Curve
NRS: Numerical Rating Scale
ITT: Intention-to-treat
PONV: Postoperative Nausea and Vomiting
